# The Healthcare Experiences of African Americans with a Dual Diagnosis of HIV/AIDS and a Nutrition-Related Chronic Disease: A Pilot Study

**DOI:** 10.3390/healthcare11010028

**Published:** 2022-12-22

**Authors:** Meena Mahadevan, Ndidiamaka Amutah-Onukagha, Valerie Kwong

**Affiliations:** 1 Department of Nutrition and Food Studies, Montclair State University, Montclair, NJ 07043, USA; 2 Department of Public Health and Community Medicine, Tufts University, Boston, MA 02111, USA

**Keywords:** HIV, African American, nutrition, comorbidity, health

## Abstract

For HIV-positive African Americans, the mistrust of medical providers due to anticipation of unequal treatment care, prejudice, and bias can become a major deterrent to medication and treatment adherence. Although programs and services incorporate strategies to improve patient–provider relationships, a deeper understanding of their healthcare experiences, especially among those with a dual diagnosis of HIV/AIDS and a nutrition-related chronic disease, is lacking. This qualitative study aimed to address this gap by conducting focus groups with participants who identified themselves as being African American, and having a dual diagnosis of HIV/AIDS, and a chronic disease. Content analysis generated several major themes, highlighting the impact of a negative healthcare experience on their ability to self-manage their health. Factors such as lack of consistency in care team, negative interactions with doctors, feelings of stigma due to prejudice and bias from healthcare staff, loss of privacy, and the need for comprehensive services that targeted their physical, emotional, and nutritional health emerged as recurring sub-themes. These findings provide the foundation for the design of a comprehensive intervention model that helps participants to communicate their medical needs more effectively, thus optimizing their overall health outcomes and quality of life.

## 1. Introduction

It is well established that a focus on patient-centered care for people living with HIV (PLWH) is correlated with positive outcomes and health-seeking behaviors, resulting in the increased utilization of healthcare services [1]. Additionally, medical mistrust, defined as a general lack of trust toward health care services and the overall health care system, has been suggested as a factor that also unfavorably impacts patient–physician interactions, and patients’ health-related outcomes [2,3]. Studies examining perceived quality of care received among patients show that African Americans report lower levels of trust and confidence in their primary care providers due to a variety of factors including lack of access to a consistent or culturally competent healthcare team [4]. As a result, they are more likely to seek medical information from family, faith-based groups, and friends [5,6]. Factors such as underestimating their symptoms, not requesting in-depth medical information, or not prescribing medications can further lower treatment adherence rates in this multiply vulnerable and underserved group [7,8,9,10,11].

Studies show that programs designed around the individual (knowledge, attitudes and beliefs), interpersonal (social support and size of social networks), environmental (availability of relevant information and educational resources), and institutional (public health policies) constructs of the socio-ecological model can be critical to improving health outcomes because they consider the dynamics between the contextual factors, and thus, by linking individuals’ health behaviors with their unique life experiences, they assist health professionals in combating barriers to behavior change within a population more effectively [12,13,14,15,16,17].

In 2012, an intervention was designed to teach HIV-positive African American women how to better prioritize and manage their nutritional health more effectively [18,19,20]. The goal of Project THANKS (Turning HIV into New Knowledge for Sisters) is to help program recipients adopt a healthier lifestyle through a series of workshops that focus on setting a personal/lifestyle goal for better nutrition, consuming balanced meals, and learning how to self-advocate for better treatment programs and services. The intervention was developed primarily to address the chronic diseases that are prevalent in the general African American population and are estimated to be among the leading causes of non-HIV-related deaths in this ethnic group [21,22,23]. The prolonged use of highly active antiretroviral therapy, particularly with the protease inhibitors, has been associated with reduced food intake and nutrient absorption which ultimately predispose these individuals to an increased risk for overweight/obesity, diabetes, cancers, hypertension, renal, or cardiovascular diseases [24]. While some HIV medications can cause side effects such as nausea, vomiting, anorexia, and diarrhea, making food intake difficult, others are known to result in eating patterns based on an increased craving for sweets and sugary beverages and replacement of nutrient-dense foods with salty and fatty snacks, further increasing their risks for the aforementioned chronic diseases [25,26].

Each chronic disease has its own highly specific nutritional and healthcare needs. People living with multiple chronic conditions need extensive support in mastering and sustaining the complex self-care behaviors that are necessary to enable them to live as healthily as possible. The primary aim of self-care (self-monitoring of blood glucose and pressure levels, medication adherence, diet compliance, continuous adjustment of food intake and exercise, and stress management) is to prevent and minimize long-term complications, and to enhance quality of life [27]. In a recent study involving older HIV-positive African American women aged 52–65, diabetes and hypertension were perceived to be more difficult to self-manage than HIV. This difficulty was mainly attributed to a lack of adequate knowledge and skills [28].

For impoverished African Americans with a dual diagnosis of HIV and a nutrition-related chronic disease, contextual factors such as lack of access to healthy food items in their neighborhood, poverty, homelessness, discrimination, and feelings of isolation and stigma associated with using soup kitchens and other sources of free food, lack of cultural competency in health care staff, and lack of continuity in the care received due to structural barriers such as strenuous distances and inadequate transportation may all prevent them from making healthy food choices and adhering to guidelines for chronic conditions [29]. Ultimately, the combined effects of these conditions pose serious threats to their health, resulting in a larger burden of hospitalization, outpatient, and emergency room visits [30]. 

While Project THANKS incorporates strategies that are designed to motivate, empower, and improve treatment adherence rates among PLWH, the unique perspectives of those with comorbidities, the negative influences and interactions they may experience in a clinical setting, and the impact of these experiences on their overall health and quality of life have not been sufficiently examined. Thus, the present study was undertaken with the primary goal of generating findings that can help expand the curriculum of Project THANKS for both African American men and women with a dual diagnosis of HIV and a nutrition-related chronic disease. The study aimed to highlight that an understanding of how these individuals interact with their physicians and experience the healthcare system is critical to the design and integration of educational components that teach them to reduce feelings of stigma and isolation in a clinical setting and present their multiple medical needs and concerns more effectively, thereby enhancing their overall health and quality of life.

## 2. Materials and Methods

While the current study was conducted with the overall goal of refining Project THANKS [18,19,20], it has its own objectives of understanding of how individuals faced with a dual diagnosis of HIV/AIDS and chronic diseases interact with their physicians and experience the healthcare system. Therefore, the sample that was recruited for this study is different and unique to this study. 

This study utilized focus groups, which is a qualitative methodology. Purposive sampling was utilized, as it was important to ensure that participants all shared the same phenomena, which was living with HIV and being diagnosed with at least one other chronic illness. Inclusion criteria included the following: (a) English-speaking African American woman or man between ages 21 and 55 years; and (b) positive for HIV serostatus and diagnosed with an additional chronic disease such as diabetes, hypertension, or heart disease (documented by the facility staff through medical records or demonstration of prescriptions, medications, and medical appointments). 

All participants were recruited from two harm reduction agencies that are based in New Jersey: AIDS Resource Foundation for Children (ARFC), Newark, NJ, USA, and Coalition on AIDS in Passaic County (CAPCO), Paterson, NJ, USA. Both organizations offer transitional housing, case management support, referrals to medical and mental care, and vocational/educational services that are designed specifically for low-income men and women living with HIV/AIDS. 

An email explaining the purpose of the study, short-term and long-term goals, and format of the data collection procedures including the location, date and time, approximate length, and nature of questions that were to be asked during the focus group discussion was sent to the case managers at each agency. The staff helped identify a list of eligible and interested men and women that were receiving support services from the agency at the time of the study. Informed consent was obtained prior to administration of the questionnaires. The facility staff were available to read the informed consent to those participants who may have had a difficult time filling them out on their own. As per IRB approval, all participants provided both written and verbal consent and stated that they were willing to participate in the study.

Data for the study were collected from January 2018–May 2018. Approval for the study was granted by the Montclair State University Institutional Review Board. Upon arrival at the focus group location, participants were asked to review, sign, and date the informed consent forms. The consent form advised participants to note the confidentiality of their responses within the focus group setting, and to accept that they could withdraw from the study at any time without reason or loss of services from the harm reduction agency. They were also asked to complete a brief questionnaire to record their age, gender, education level, substance abuse history, and medical history. They were each given $20 in cash for their effort in participation and asked to sign a receipt form. 

A total of three focus groups were conducted in Newark and Paterson NJ, USA: one group at ARFC (all women) and two groups at CAPCO (one group with all women, and the other group with all men). All the focus groups were held in private rooms at the agencies, lasting approximately 75–90 min. The first 15 min of the focus group sessions were used by the facilitators to introduce themselves, and to allow participants to ask any last-minute questions. Additionally, all the sessions were adjourned for a 15-min break after the first 30 min, to allow participants to use the bathroom, grab water or snacks, or to simply stretch. After the break, the sessions resumed. Therefore, the actual length of the sessions that included active discussions with participants was approximately 45–60 min long. With the participants’ permission, the sessions were audiotaped. One of the members of the research team took on the role of being the lead facilitator/moderator for the discussions. A graduate assistant, who was trained to observe and take notes on any side conversations that may not have been fully captured during the discussions, was also present. These observations and notes helped to point out any topics or questions that needed to be followed up with the entire group in order to triangulate the findings. The focus groups were all facilitated and led by the same facilitator and graduate assistant. 

A semi-structured focus group guide was used to facilitate the discussions. The guide was developed based on pilot data results, which have been collected and reported previously. The questions were divided into four domains: (1) attitudes and beliefs about health, (2) relationships with their physicians, (3) facilitators and barriers to quality care, and (4) the need for any resources and support in navigating the healthcare system. Sample questions for each category are listed in Table 1. Due to the complexity and sensitivity of the topic, questions were in-depth and semi-structured in format to allow for open discussion that pertained to the topic in addition to giving participants control of the session. 

The audiotapes from the three focus groups were transcribed verbatim and analyzed for major themes using content analysis techniques in Microsoft WORD, version 15.34. As a first step, a research assistant developed a draft of the codebook based on the questions and prompts used in the focus group guide as well as constructs from the socio-ecological model. The first and second authors reviewed the codebook as a strategy to ensure that the codes would allow for a comprehensive retrieval and organization of the focus group transcripts into common trends and patterns. The final set of codes was assigned to passages of text throughout each transcript by the research assistant, who was then able to group all coded text into themes and sub-themes. One of the steps of this grouping procedure involved comparing different people (such as their views, situations, actions, accounts, and experiences) within the same focus group, and comparing data across the three focus groups. This process helped attain data saturation and generate a set of themes that was grounded in the participants’ own lived experiences.

In order to enhance the trustworthiness of the findings, the first and second author independently analyzed the coded transcripts and met frequently to cross check their interpretations of the data. They each made a note of the regularities, patterns, and explanations in the transcripts and came to a shared conclusion on the intended and implied meaning of the participants’ words. The goal was to gain new perspectives and insights with as little bias or emotional involvement as possible. Thus, a willingness on the researchers’ parts to discard preconceived notions that conflicted with the data, and a desire to represent the multiple views of the research participants in an honest and thorough manner helped this research remain free of personal subjectivity. Responses from the demographic questionnaires were entered into Microsoft EXCEL, version 15.34, and analyzed to obtain a basic demographic profile of the participants.

## 3. Results

A total of 26 HIV-positive African American men and women participated in the study (Table 2). The majority were women (61.5%). The mean age of the group was 53 years. All participants (100%) reported a past or current history of substance abuse, and mental health disorders such as depression and/or anxiety. Most reported being high school graduates/GED recipients (46.1%), or having some high school education (30.7%), being single (61.5%), homeless (88.4%), and unemployed or laid-off (88.4%). All the participants reported a diagnosis of a nutrition-related chronic disease such as hypertension (57.7%), diabetes (30.8%), or heart disease (11.5%). Except for one participant, all indicated currently seeing a physician, and taking prescribed medications for the reported condition.

Content analysis of the focus group transcripts generated three major themes, and several sub-themes highlighting their overall healthcare experiences, the stressors contributing to any negative experiences, and strategies adopted by participants to cope with these experiences. Below we provide a brief description of each theme, accompanied by the most representative quote under each theme/subtheme to illustrate our interpretation. For confidentiality purposes, participants’ actual names have not been used. 

### 3.1. Theme 1: Negative Interactions

The focus group discussions began by asking participants to describe their overall experience with the healthcare system, and more specifically, to reflect on how the types of interactions/relationships they had with their physicians contributed to this experience. They were encouraged to give examples to demonstrate their point. The importance of establishing a bond with their physicians, and building a relationship based on honesty, respect, and trust, emerged as a prominent and recurring theme in all three focus groups. For some, not having a consistent physician or team of professionals appeared to cause frustration, and ultimately impacted their ability to be a more active participant during the treatment process:

*“You know, you build that bond, but when you get someone different every time … And I hate needles, so they just take my … I be frustrated every time I go. ‘Cause I know I gotta give blood. I don’t like to give blood. I feel as though for somebody that I knew before might know a little bit more about me from the last visit”.*

Some individuals talked about instances wherein they felt that their physician did not seem more personally invested in their care:

*“I would like for them to actually get to know who I am and what I am going through instead of signing off a script and pushing me out the door”.*

Not being provided with an explanation of the care process seemed to have caused considerable stress and confusion for some:

*“And some doctors they’ll push you off and say, “Oh, I recommend you go here. Or I recommend you” … You know what I mean? They the same type of doctor you are. Why are you sending me there? I don’ been sent from Bergen Country to Newark to Edison, NJ, and everybody go, “I suggest you go back to Newark University.” And there, all they wanna do is give me some type of chemo treatment. Chemo for what? I ain’t got no cancer. Then they hit you with “Oh, well, your immune system is hyped up and it’s attacking your eyes”.*

Some participants described interactions that included poor communication skills and language barriers:

*“I asked her, and she looked at my chart, she said, “You lucky your ass is alive.” Okay. Now she’s supposed to be the smartest one on the staff. Really? No bedside manners”.*

Only one participant provided an example of a positive interaction, which seemed to contrast with all the aforementioned experiences:

*“I have a very good rapport with my doctor, for the simple fact that I’ve been seeing him since 1998. He talks so much to me, I have to tell him I gotta go, but there’s nothing that we don’t discuss. Anything that I feel like I need to say to him, I will say it. And he’s … We sat up and even talked about diets, food that should be eaten and what not. Mind you, he’s not a nutritionist, but we cover everything. Before he place me on a medication for whatever is going on with me, he’ll do his research and say I’m not gonna put you on this right now. Let me do my research on it”.*

### 3.2. Theme 2: Barriers to Quality Healthcare

An animated discussion ensued in all three focus groups when participants were asked to describe any concerns/barriers that they faced while navigating their healthcare experience. These barriers ranged from insurance coverage issues to feeling stigmatized and threatened about losing their privacy due to confidentiality violations. For instance, the sentiment of lacking adequate insurance coverage to obtain needed medications was echoed by nearly everyone in the focus groups:

*“People are dying because they do not have health insurance. How can we bury some of these people? With HIV? With AIDS? Medicine is very important. I had to bury two of them because they didn’t have insurance”.*

One individual commented on how she felt forced to consume over the counter instead of prescribed medications:

*“Mine [insurance plan] ain’t good, I got well-care…My doctor order me Motrin but because of the well-care, I got the cheap, fake Motrin … I have to take like 5 pills because its 400 milligrams. That is what my insurance is paying for”.*

Feeling underrepresented, and discriminated against due to their ethnic minority, socioeconomic, or HIV status also emerged as a recurring sub-theme during the discussion of any perceived concerns/barriers to their healthcare experiences:

*“They pushed it [improvements in healthcare for HIV-positive people] under the rug. They didn’t care because it was doing damage to US. Now they pay attention because it is happening to rich White people, now they care…Now it is all over the news, but when it was right here in our community. It wasn’t like they weren’t paying attention”.*

Feeling stigmatized and threatened about loss of privacy due to confidentiality violations were recurring sub-themes throughout the three focus groups. Several participants narrated specific incidents in clinical settings, wherein they felt that they were being treated differently because of their HIV status:

*“If you go to the emergency room or places like that, if you see a doctor … You go in there for a cold or a cut on your finger, and you see somebody come in there with a mask or line all over they face, tied up, what do you think that is? You think that’s not showing you that it still exists? They look at your blood and see HIV or AIDS anything, they start masking up. Like you got something that’s contagious through the air. I feel judged, I have to put my walls up”.*

Some individuals went on to describe specific interactions which led to a perception that the particular physician lacked compassion, or was not qualified to treat their condition:

*“And it’s sad because a lot of even the health care professionals are ignorant. They are not educated enough to deal with HIV patients. Because the doctors should know. But some get educated, some don’t … stigma is alive and well”.*

It appeared from a few participants’ comments that such interactions were indeed barriers to receiving quality care as they ultimately negatively impacted treatment adherence:

*“I have a condition where my blood clots up. And then I have a blockage in my artery. And my other primary doctor, she sent me to a clinic to get these tests and once they seen HIV, I could see the difference, you know? And then I see them putting on gloves. And I’m looking around the room like, “Okay, that’s how you feeling? That was in my head. So I took the test but I never went back”.*

For one individual, the confidentiality issues appeared to stem from the lack of consistency in care team:

*“After I tell them all of my business, two weeks later there’s someone new. I have to start all over again with a stranger…That is where confidentiality goes out the door”.*

A couple of the comments suggested that the confidentiality violations had contributed to an undermining of trust in the healthcare system:

*“Doctors putting my business out there makes me want to fall back on poor behaviors. No confidentiality. I changed doctors. I did not trust her anymore. You have to be careful with what you allow certain people and things to have access to”.*

### 3.3. Theme 3: Needs and Advocacy

A major theme that emerged during the discussions pertained to the types of support and resources needed to improve their healthcare experiences, especially as it relates to being diagnosed with multiple chronic diseases. For instance, many participants talked about struggling to manage the side-effects associated with medications, and learning to cope with the impact on their weight, eating habits, and overall health:

*“Because as long as you taking your medication as prescribed for your HIV, that’s going to take care of that, okay, but if you have other illnesses going on, it’s the HIV is not going kill you, it’s the high blood pressure, diabetes is not only from HIV. It’s just, it’s, we have to be more focused on it than someone that doesn’t have the virus”.*

Mental health issues such as depression and anxiety, and the negative toll of these conditions on their physical, social, and emotional health, were also discussed in all three focus groups:

*“Because I can remember five years ago, being diagnosed. Like I didn’t know what to do. I’m not from Patterson, I come all the way to Patterson to come to treatment to find out I’m sick. So it’s like, “Who do I tell? Like should I call my kids, because my kids don’t know about me.” I don’t have parents, I can’t call them. The person I caught it from, she’s been active using. So it’s like, who do you tell?”*

Some talked about the need for expert help and services to help them stay connected to family, friends, and other HIV-positive individuals:

*“I feel like my care… could do better if I actually had a psychologist who actually took the time to help me understand … things I’m going through instead of trying to push medications on me”.*

In responding to the question on addressing/advocating for better healthcare, participants were divided on who should best represent their needs. Some opined that having healthcare professionals or researchers who are familiar with their needs advocate for better policies and programs would garner more attention to their conditions, and thus, have a greater impact:

*“Protesting can help but it only gets so far … Your voice gets heard for a little bit, they come and pacify you and then move on. When people like you [the researchers] come to ask us questions, I think you should be our voice. I think they would pay more attention to you than people like us”.*

In contrast, some individuals felt strongly about self-advocacy for the simple reason that it resulted in being a more educated and active participant in one’s own healthcare:

*“A lot of people is they own worst enemy. They scared of everything. I wanna say that you need to be the best advocate for yourself. Listen, if you’re unhappy, you need to speak up and have changes made.”*

## 4. Discussion

This study explored the issue of healthcare experiences among African American men and women living with HIV/AIDS who were diagnosed with an additional nutrition-related chronic disease such as diabetes, hypertension, or heart disease. The goal of this research was to generate findings that can help inform the development of new, or making modifications to, existing educational components of Project THANKS, an intervention that was originally designed to help HIV-positive African American women manage their HIV-related healthcare needs more effectively. Using a patient-centered perspective approach to expand the curriculum with educational components that target both men and women faced with a dual diagnosis presents an integral opportunity to keep all PLWH stay better connected to quality care and services. Through the focus groups, this pilot study has helped provide a foundation for further investigating the healthcare challenges reported by the participants. 

The use of qualitative methodology is especially useful for research that is designed with the purpose of eliciting information that may not be easily identified using surveys or questionnaires. For example, they provide an opportunity for participants to explain, in their own words, how and why they arrive at the choices they make [31]. The focus groups have the added advantage of creating a comfortable environment where individuals are able to share their feelings openly with one another, thus forming a stronger support system amongst themselves [32,33,34]. The purposive sampling technique used in the study was also particularly beneficial in highlighting the unique experiences shared by a distinct group of individuals faced with the dual diagnosis of HIV/AIDS and one or more chronic diseases. 

A content analysis of the three focus group discussions generated several major themes that provide the necessary foundation for making some important modifications to Project THANKS. For instance, the inability to develop a bond with and trust their physicians to provide quality care emerged as a major theme throughout all three focus groups. Lack of consistency in healthcare team, stigma, prejudice, and lack of empathy were identified as contributing factors. The curriculum for Project THANKS already includes a lesson plan that encourages its recipients to examine existing relationships with their partners, friends, or family members. The goal is to have them realize that an important part of pursuing a healthy lifestyle is to develop friendships and relationships with healthier boundaries. In keeping with evidence-based recommendations, the modified curriculum will include additional role-playing and case scenario activities which will allow them to openly express feelings of stigma and communicate their needs with their physicians more effectively [35,36,37]. Intervention experts also caution, however, that efforts to improve patient–provider relationships should be directed at everyone within a healthcare facility, including patients, physicians, nurses, guards, cleaners, and administrative staff [38,39]. Therefore, in keeping with this recommendation, Project THANKS will also incorporate a lesson plan and/or develop print materials for distribution that are specifically designed to help organizational staff at all levels across the organization understand the impact of stigma, bias, and prejudice on adding to the burden of mental health issues, and possibly contributing to lower treatment adherence rates.

Feeling overwhelmed from managing specific treatment protocols associated with a nutrition-related chronic disease, in addition to HIV/AIDS, emerged as a recurring theme as well. The need to juggle multiple symptoms, managing medication side-effects, scheduling visits with multiple specialists to track biomarkers, not having the knowledge to plan a balanced meal that considers individual preferences, allergies, and cultural backgrounds, and not having adequate access to an integrated healthcare team were all described as challenges that added to their physical and mental health burden. The curriculum for Project THANKS will be modified to include additional evidence-based strategies that will help these individuals navigate the trajectory of dual diagnosis more seamlessly. For example, in addition to the existing emphasis on the importance of making a lifestyle change for better management of HIV/AIDS, the workshops will focus on teaching individuals how to purchase, prepare, and consume a balanced diet that was aligned with the dietary principles for a specific comorbid condition, incorporate physical activity into their daily schedule, and schedule timely medical/laboratory/mental health appointments to track symptoms that may interfere with their ability to self-manage [40,41].

Finally, the need for additional support services that help them better cope with feelings of depression, anxiety, and isolation brought on by stigma, bias, and discrimination was a prevalent theme in all three focus groups. Although Project THANKS does not currently provide mental health services through its intervention, the curriculum recognizes the need for educational components that help program recipients to monitor and manage symptoms of mental health disorders [42,43,44,45,46,47]. The curriculum will partner with trained facilitators who can help program recipients learn and adopt various stress management strategies such as meditation, exercise, and reaching out to members of their social network. 

The authors acknowledge that there are some limitations to this study. The sample consisted entirely of clients who were recruited from two harm reduction agencies in New Jersey. Therefore, the experiences of these NJ-based clients may not be representative of all HIV-positive individuals across the United States. Secondly, the technique of purposive sampling was used to select a sample consisting of HIV-positive clients that shared a specific set of demographic characteristics. While this helped to shed light on how this group navigates their unique healthcare needs, the sampling technique nevertheless limits the external validity of the study’s findings. The major themes that emerged from the focus group discussions in this study may not be generalizable to others that do not share a similar demographic profile. Despite the limitations to generalizability posed by the purposive sampling technique used in this study, the findings nevertheless help provide preliminary data on an issue that is worth investigating further in subsequent research. While we recognize that there may be within-group similarities and differences that are not accounted for in our sample, the themes generated in this research have contributed to creating an improved model of care that allows for meaningful and sustainable changes that can potentially benefit the entire HIV/AIDS community.

## 5. Conclusions

Despite the limitations to generalizability posed by the purposive sampling technique used in this study, the findings nevertheless help provide some preliminary data for practitioners that are directly involved in the care of African American men and women living with a dual diagnosis of HIV/AIDS and one or more nutrition-related chronic diseases. Not having access to a physician or a team of professionals that anticipates stigma, bias, and discrimination, and accordingly advocate for them can result in patient distrust, and in turn, resistance in sustaining treatment. A continued examination of how these individuals perceive their healthcare experiences is critical to the design of comprehensive intervention models that can optimize health outcomes and improve their overall quality of life.

## Figures and Tables

**Table 1:** Focus Group Guide.

Category	Sample Questions
Attitudes and Beliefs About Health	1. How would you describe the health care that you are currently receiving? 2. Have you had any negative experiences with seeking health care? Give an example. 3. Why do you think you had that negative experience? 4. Have you had any positive experiences with health care? Give an example.
Relationships with Physicians	5. What comes to mind when you think about the relationship you have with your doctor? 6. What do you look for in selecting a doctor? 7. In your opinion what makes someone a good doctor? 8. How did this influence your experience?
Facilitators and Barriers to Quality Care	9. What is your biggest concern or barriers that you face regarding your health care? Give an example. 10. In your opinion, what are some of the things that your health care provider/doctor/nurse can do to address these barriers?
Needs and Advocacy	11. In your opinion, what are some of the things that you can do to improve your healthcare? (Prompt: specific communication techniques with healthcare professionals, tasks, actions such as making priority lists, goal setting, stress management, eating better, getting enough sleep, etc.) 12. What is the role of the media/local politicians (mayor, governor, others in local/state/federal office) in helping to address/advocate for better healthcare for people diagnosed with HIV/AIDS and other chronic diseases such as diabetes, hypertension or heart disease? What are the ways in which this should be done? 13. What is your role in advocating for better healthcare for yourself? What are the ways in which this should be done?

**Table 2:** Demographic Profile.

**Sex**	
Female	*n* = 16 (61.5%)
Male	*n* = 10 (38.5%)
**Age**	Mean = 53 years, SD ± 10.8
**History of past or present substance abuse**	*n* = 26 (100.0%)
**Education Level**	
Less than High School	*n* = 4 (15.3%)
Some High School, No GED	*n* = 8 (30.7%)
High School Graduate or GED	*n* = 12 (46.1%)
Some College	*n* = 1 (3.8%)
B.A./B.S	*n* = 1 (3.8%)
Associates Degree	*n* = 0 (0.0%)
Graduate Degree	*n* = 0 (0.0%)
**Marital Status**	
Single	*n* = 16 (61.5%)
Long-term relationship	*n* = 8 (30.7%)
Married	*n* = 1 (3.8%)
Separated	*n* = 1 (3.8%)
Divorced	*n* = 0 (0.0%)
**Living Situation**	
House/apartment	*n* = 2 (7.6%)
Homeless (in a shelter)	*n* = 23 (88.4%)
Group home/halfway house	*n* = 1 (3.8%)
Don’t know/refused	*n* = 0 (0.0%)
**Employment Situation**	
Unemployed or laid-off	*n* = 23 (88.4%)
Working full-time	*n* = 1 (3.8%)
Working part-time	*n* = 1 (3.8%)
Don’t know/refused	*n* = 1 (3.8%)
**Chronic Conditions (in addition to HIV/AIDS)**	
Diabetes	*n* = 8 (30.8%)
Heart Disease	*n* = 3 (11.5%)
Hypertension	*n* = 15 (57.7%)
**History of past or present mental health conditions such as depression and/or anxiety**	
Yes	*n* = 26 (100.0%)
No	*n* = 0 (0.0%)
**Currently Seeing Doctor/Nurse for Chronic Condition**	
Yes	*n* = 25 (96.1%)
No	*n* = 1 (3.8%)
**Currently Taking Prescribed Medication for Chronic Condition**	
Yes	*n* = 25 (96.1%)
No	*n* = 1 (3.8%)

## Data Availability

Not applicable.
